# Supplementary material for: End Sequence Analysis Toolkit (ESAT) expands the extractable information from single-cell RNA-seq data
Source: Genome Res. 2016 Oct;26(10):1397–410. doi: 10.1101/gr.207902.116 (PMC5052061; doi:10.1101/gr.207902.116)
Supplement: Supplemental Material [file supp_gr.207902.116_Supp_Materials.tar.gz › ESAT-esat_v0.1/doc/html/classbroad_1_1pda_1_1differential_expression_1_1_f_w_e_r_distribution.html]

ESAT: broad.pda.differentialExpression.FWERDistribution Class Reference


ESAT


- Main Page
- Related Pages
- Packages
- Classes
- Files

- Class List
- Class Index
- Class Hierarchy
- Class Members

All Classes Namespaces Files Functions Variables Enumerator Pages

- broad
- pda
- differentialExpression
- FWERDistribution

Public Member Functions |
Public Attributes |
List of all members

broad.pda.differentialExpression.FWERDistribution Class Reference

Collaboration diagram for broad.pda.differentialExpression.FWERDistribution:

[legend]

|  |  |
| --- | --- |
| Public Member Functions | |
|  | FWERDistribution (double[] observed, Matrix permutations) |
|  | |
| double | getFWER (double d) |
|  | |

|  |  |
| --- | --- |
| Public Attributes | |
| EmpiricalDistribution | minDist |
|  | |
| EmpiricalDistribution | maxDist |
|  | |

## Constructor & Destructor Documentation

|  |  |  |  |
| --- | --- | --- | --- |
| broad.pda.differentialExpression.FWERDistribution.FWERDistribution | ( | double[] | *observed*, |
|  |  | Matrix | *permutations* |
|  | ) |  |  |

## Member Function Documentation

|  |  |  |  |  |  |
| --- | --- | --- | --- | --- | --- |
| double broad.pda.differentialExpression.FWERDistribution.getFWER | ( | double | *d* | ) |  |

## Member Data Documentation

EmpiricalDistribution broad.pda.differentialExpression.FWERDistribution.maxDist

EmpiricalDistribution broad.pda.differentialExpression.FWERDistribution.minDist

---

The documentation for this class was generated from the following file:

- java/broad/pda/differentialExpression/FWERDistribution.java


---

Generated on Fri Aug 15 2014 15:10:59 for ESAT by  

 1.8.7
